# Presphenoidal synchondrosis fusion in DBA/2J mice

**DOI:** 10.1007/s00335-012-9437-8

**Published:** 2012-11-21

**Authors:** Allysa Adams, Brandeis McBratney-Owen, Brittany Newby, Margot E. Bowen, Bjorn R. Olsen, Matthew L. Warman

**Affiliations:** 1 Orthopaedic Research Laboratories, Boston Children’s Hospital, Boston, MA USA; 2 Present Address: University of Melbourne, Melbourne, VIC Australia; 3 Department of Genetics, Harvard Medical School, Boston, MA USA; 4 Harvard School of Dental Medicine, Boston, MA USA; 5 Howard Hughes Medical Institute, Boston Children’s Hospital, Boston, MA USA

## Abstract

Cranial base growth plates are important centers of longitudinal growth in the skull and are responsible for the proper anterior placement of the face and the stimulation of normal cranial vault development. We report that the presphenoidal synchondrosis (PSS), a midline growth plate of the cranial base, closes in the DBA/2J mouse strain but not in other common inbred strains. We investigated the genetics of PSS closure in DBA/2J mice by evaluating F1, F1 backcross, and/or F1 intercross offspring from matings with C57BL/6J and DBA/1J mice, whose PSS remain open. We observed that PSS closure is genetically determined, but not inherited as a simple Mendelian trait. Employing a genome-wide SNP array, we identified a region on chromosome 11 in the C57BL/6J strain that affected the frequency of PSS closure in F1 backcross and F1 intercross offspring. The equivalent region in the DBA/1J strain did not affect PSS closure in F1 intercross offspring. We conclude that PSS closure in the DBA/2J strain is complex and modified by different loci when outcrossed with C57BL/6J and DBA/1J mice.

**Electronic supplementary material:**

The online version of this article (doi:10.1007/s00335-012-9437-8) contains supplementary material, which is available to authorized users.

## Introduction

**Fig. 1:**
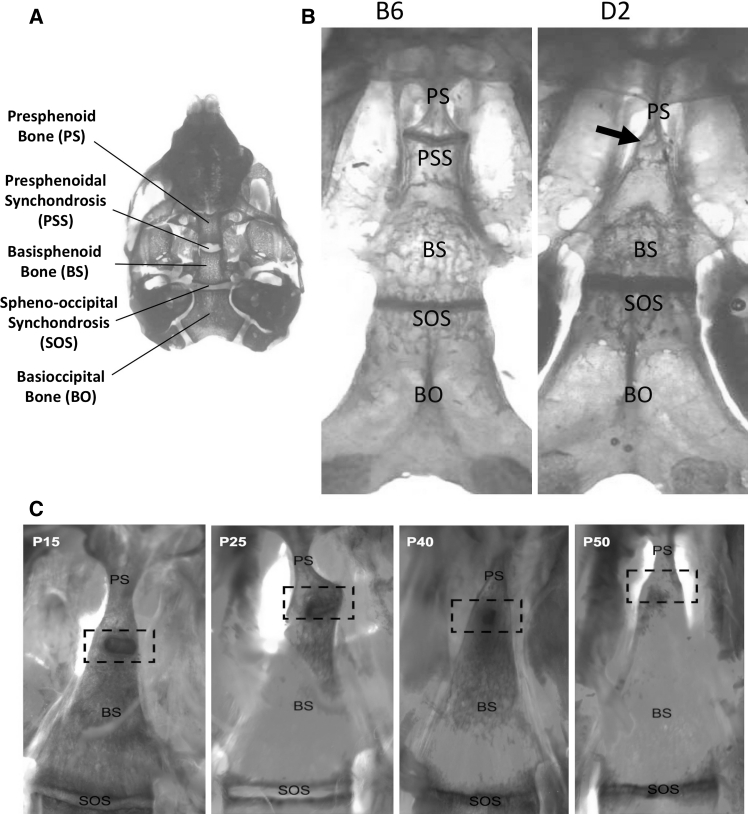
Closure of the presphenoidal synchondrosis occurs in the DBA/2J inbred strain. **a** Photograph of a newborn (P1) C57BL/6J cranial base (shown from a superior view after the brain was removed) that had been stained with Alcian blue and Alizarin red to indicate cartilage and bone, respectively. Important landmark structures are noted; the presphenoid synchondrosis (PSS) is located between the presphenoid (PS) and basisphenoid (BS) bones. **b** Photographs of adult cranial bases, stained with Alcian blue and Alizarin red, demonstrating a symmetric and patent structure in C57BL/6J and a closed PSS in DBA/2J (*arrow*). **c** Cranial bases of DBA/2J mice at different postnatal ages stained with Alcian blue and Alizarin red. The *dashed box* indicates the location of the PSS. The P15 specimen demonstrates bilateral bony fusion across the PSS. The P25 specimen demonstrates unilateral bony fusion with resultant angular deformity. Bilateral fusion with residual cartilage is seen in the P40 specimen, whereas the cartilage is absent in the P50 specimen (Color figure online)

**Fig. 2:**
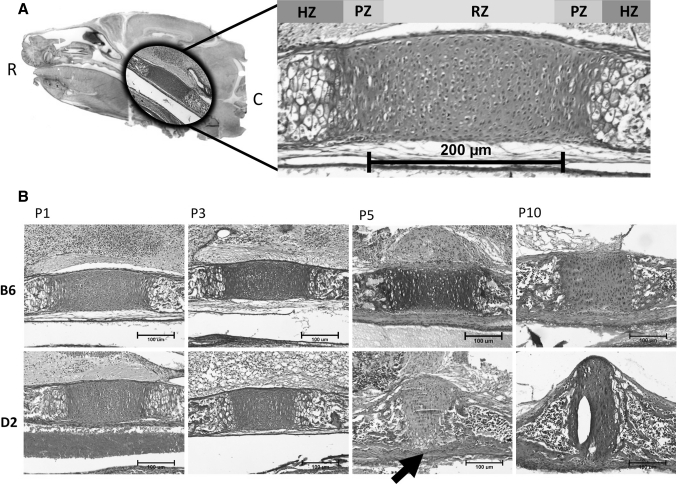
Histologic analysis of PSS closure in DBA/2J mice. **a** Photograph of a midline sagittal section through a P1 C57BL/6J mouse cranium; a photomicrograph within the oval contains an enlargement of the area with the PSS superimposed. Photomicrograph on the right is from a C57BL/6J P1 mouse with the PSS in a rostral (R)–caudal (C) orientation. The bilaterally symmetric resting zone (RZ), proliferating zone (PZ), and hypertrophic zone (HZ) chondrocyte-containing regions are indicated. *Scale bar* 200 μm. **b** Photomicrographs of midline sagittal sections through the PSS of C57BL/6J (B6) and DBA/2J (D2) mice at 1, 3, 5, and 10 days after birth (P1, P3, P5, P10) stained with hematoxylin and eosin. C57BL/6J and DBA/2J mice have similar looking PSS at P1 and P3, although the rostral–caudal length consistently appears shorter in DBA/2J than in C57BL/6J mice. By P5, the PSS in the DBA/2J mouse has lost rostral–caudal symmetry; instead, the HZ is at the ventral surface (*arrow*) and the RZ bulges from the dorsal surface. At P10, the PSS in the DBA/2J mouse lacks recognizable RZ, PZ, and HZ regions, whereas the RZ region is readily seen in the C57BL/6J PSS. *Scale bars* 100 μm (Color figure online)

The synchondroses of the mammalian cranial base are important centers of longitudinal growth in the skull, playing a critical role in both the proper anterior placement of the face and the development of a normal cranial vault (Bjork 1955; Ford 1958; Rosenberg et al. 1997). The midline of the mouse cranial base contains two growth plates, the spheno-occipital synchondrosis (SOS) and the presphenoidal synchondrosis (PSS) (Fig. 1a). In mice, these synchondroses contribute to growth of the occipital and sphenoid complex after birth. Unlike in humans, whose growth plates eventually close, murine synchondroses remain cartilaginous even after anteroposterior growth has ceased. Structurally, synchondroses in both humans and mice are arranged as two bilaterally symmetric growth plates, each containing chondrocytes at stages of differentiation that can be distinguished by morphology and/or gene expression (Fig. 2a). In the growth plates of long bones, chondrocyte organization and differentiation are tightly regulated processes dependent on both systemic and local mediators (Burdan et al. 2009).

Studies in humans and mice have identified genes and pathways that affect growth and maintenance of synchondroses. In humans, mutations in TWIST and in FGF signaling pathway components have been identified in patients with craniosynostosis (Gripp et al. 2000; Nuckolls et al. 1999; Wilkie 1997), and missense mutations affecting TGFβ are associated with a variety of craniofacial abnormalities (Loeys et al. 2006). Mice with alleles equivalent to the human mutations also exhibit abnormal synchondrosis closure (Bourgeois et al. 1998; Matsushita et al. 2009; Wang et al. 2005; Yin et al. 2008). Conditional inactivation of other pathways in mice reveals roles for Indian hedgehog (Young et al. 2006) and primary cilium-based signaling (Kolpakova-Hart et al. 2008; Koyama et al. 2007; Ochiai et al. 2009). Finally, mechanical stress has been shown to affect SOS patency (Lei et al. 2008).

Here we report that the DBA/2J inbred mouse strain exhibits PSS closure, whereas other common inbred strains do not. We describe the histologic changes that accompany PSS closure in this strain and the pattern of inheritance of PSS closure when DBA/2J mice are outcrossed with C57BL/6J and DBA/1J mice. Finally, we show that a locus on chromosome 11 strongly influences PSS closure in the DBA/2J × C57BL/6J cross but not in the DBA/2J × DBA/1J cross.

## Materials and methods

### Mouse strains

This work was approved by the Institutional Animal Care and Use Committees at Children’s Hospital Boston and Harvard School of Dental Medicine. Inbred mice of the DBA/2J (stock number 000671), DBA/1J (000670), and C57BL/6J (000664) strains were obtained from The Jackson Laboratory (http://www.jax.org).

### Generation of F1, F1 backcross, and F1 intercross offspring

DBA/2J mice were crossed with C57BL/6J or DBA/1J mice to produce F1 offspring. Sires and dams came from both parental strains to control for parent-of-origin effects. Male and female F1 mice were intercrossed to produce F2 offspring. In addition, F1 offspring from the DBA/2J × C57BL/6J cross were bred to DBA/2J mice to produce F1 backcross offspring.

### Visual classification of PSS closure

Following euthanasia, each mouse had its cranium removed. The calvarium and brain were discarded and the skull base was either immediately visualized under the dissecting microscope or visualized following storage in 70 % EtOH. Mice were classified as having PSS closure if they had superior protrusion of the PSS cartilage, angular deformity of the PSS, or unilateral or bilateral bony bridging across the PSS. Mice were classified as having an open PSS if a nondeformed strip of cartilage spanned the boundary between the presphenoid and the basisphenoid bones. Cranial bases were examined in DBA/2J mice at postnatal day 6 (P6), P15, P21, P25, P40, P50, P61, P66, P80, and P180 (≥3/age). In addition, 82 DBA/2J mice 180 days or older were examined. The cranial bases of F1, F1 intercross, and F1 backcross offspring were examined either at the time of weaning (P21) or at P35.

### Histologic evaluation of the PSS

Crania from DBA/2J and C57BL/6J mice were recovered at P1, P3, P5, P10, and P15 (*N* = 3 per time point and strain), decalcified, dehydrated, cleared in xylene, and then mounted in paraffin. Seven-micron sagittal sections were obtained through the PSS and SOS. Slides were cleared in xylene, rehydrated, soaked in Gill Modified Hematoxylin Harlecohematoxylin (Harleco), Clarifier 2 (Richard-Allan Scientific), Bluing reagent (Richard-Allan Scientific), and Eosin Y (Richard-Allan Scientific), followed by dehydration and a final clearing in xylene. Slides were mounted with coverslips, viewed on a Leica DMLb microscope, and imaged using the OpenLab software suite. Measurement of the length of the PSS was performed digitally using the ImageJ software suite: for each animal, a measurement was taken spanning the midline of the PSS in two adjacent sections, giving a total of six measurements per mouse line per time point.

### Whole-mount staining

For Alcian blue/Alizarin red staining of adult mouse crania, the vault and brain were removed prior to fixing overnight in 100 % EtOH. Cartilage was stained overnight in 80 ml of 95 % EtOH, 20 ml glacial acetic acid, and 15 mg Alcian blue (Sigma). After washing twice in 95 % EtOH and soaking in 2 % KOH in water for 3 h, bone was stained in 1 % KOH, 7.5 mg/ml Alizarin red S (Sigma) in water overnight. Specimens were destained in 20 % glycerol, 1 % KOH in water for 2 days and then put in 20 % glycerol in water, changed daily for 5 days. Finally, crania were placed in 20 % glycerol, 20 % EtOH overnight and then stored and photographed in 50 % glycerol, 50 % EtOH.

### DNA extraction, genotyping, and linkage analysis

DNA was extracted from tail or liver using the Qiagen DNeasy Blood and Tissue Kit (Qiagen, Valencia, CA) following the manufacturer’s protocol. Whole-genome SNP genotyping was performed at the University of Toronto using the Illumina Bead Array mouse genotyping platform (Illumina, Inc., San Diego, CA), which contains 874 informative SNPs between the DBA/2J and C57BL/6J strains. Because no common region of homozygosity was identified among the F1 intercross offspring that had PSS closure, we analyzed each SNP for deviation from the expected 1:2:1 distribution by χ^2^ analysis. SNPs yielding χ^2^-derived *p* values <5 × 10^−5^ (i.e., 0.05/874 in order to correct for the multiple testing) were considered significant. Chromosome 11 microsatellite markers *D11Mit62* (5.78 cM), *D11Mit78* (10.44 cM), *D11Mit109* (23.57 cM), and *D11Mit111* (31.97 cM) were genotyped in 67 additional F2 offspring that had closed PSS and in more than 102 F2 offspring that had open PSS. The microsatellites were also genotyped in F1 backcross offspring that had closed PSS (*N* = 43) and open PSS (*N* = 38). DNA for PCR-based genotyping was obtained from tail snips using the HotSHOT protocol (Truett et al. 2000). SNP genotyping in F2 offspring was performed by PCR amplification followed by digestion with restriction enzymes that distinguish DBA/1J amplimers from DBA/2J amplimers. χ^2^ analysis was used to analyze allele distributions for the PCR-amplified microsatellite and SNPs.

### Low-coverage whole-genome sequencing and SNP detection

Genomic DNA from two female DBA/1J mice was extracted using the Qiagen DNeasy Blood and Tissue Kit and equal amounts of DNA were then pooled. Similarly, equal amounts of DNA from nine P35 F2 progeny with open PSS from the DBA/1J × DBA/2J cross were pooled. For each DNA pool, 4 μg of DNA was sheared to an average size of 200 bp using Adaptive Focused Acoustics following the manufacturer’s protocol (Covaris, Inc., Woburn, MA). The DNA fragments were then blunt-ended, 5′ phosphorylated, A-tailed, and ligated to adaptors as previously described (Bowen et al. 2011). Phusion High-Fidelity DNA polymerase (Finnzymes, Thermo Scientific, Waltham, MA) was used to amplify 12 μl of the library, in a total of five 50-μl PCR reactions, using the post-capture primers (Bowen et al. 2011). Eight cycles of PCR were performed. The resulting library was 100-bp single-end sequenced on one lane of an Illumina HiSeq2000.

Sequencing reads were aligned to the mouse reference genome (mm9) using BWA (Li and Durbin 2009), and PCR duplicates were removed using Picard (http://picard.sourceforge.net/). The DBA/2J aligned sequence was downloaded from the Welcome Trust Sanger Institute (http://www.sanger.ac.uk/). The known SNPs between the DBA/1J and DBA/2J strains were downloaded from the Mouse Phenome Database (http://phenome.jax.org/). SNPs imputed to differ between DBA/1J and DBA/2J were downloaded from the Mouse HapMap Imputed Genotype Resource (http://mouse.cs.ucla.edu/mousehapmap/beta/index.html/). Custom perl scripts were used to determine whether the known and imputed SNPs were supported by the DBA/1J and DBA/2J whole genome sequencing (WGS). Only sites covered by at least one read in each strain were considered, and for sites covered by more than one read, only homozygous sites were considered. To identify novel SNPs, variant calling was performed using SAMtools/BCFtools (Li et al. 2009) and SNPs were filtered using the GATK (DePristo et al. 2011). SNPs were selected if they had at least two reads representing the nonreference genome allele, a quality score >30, a mapping quality score >40, a combined read depth (DBA/1J + DBA/2J) >4 but <80, and did not lie in repetitive sequences (defined by RepeatMasker) or within a cluster of more than three SNPs per 10-bp window.

## Results

### The PSS does not remain patent in all inbred strains of mice

During the course of studying cranial base morphology in adult mice (6 months old/P180 or greater) from several commonly used inbred mouse strains (C57BL/6J, C3H, Balb, A/J, Nu, 129, and DBA/2J), we noticed that the PSS remained patent in every strain except for DBA/2J (Fig. 1b). The penetrance of PSS closure was 98.8 % (81/82 mice) in DBA/2J mice at P180. In order to determine when the PSS closes in this strain, we studied DBA/2J mice at different ages. By dissecting microscopic inspection we could detect unilateral or bilateral ossification at the lateral edges of the PSS as early as P6 (results not shown). By P15 DBA/2J mice exhibited lateral ossification across the PSS, which was associated with a dorsal projection of the remaining PSS cartilage (Fig. 1c). PSS fusion was nearly complete by P50 (Fig. 1c).

### The histological appearance of the PSS is different between the DBA/2J and C57B/6J strains

We examined hematoxylin and eosin-stained sagittal sections of the cranial bases of DBA/2J and C57BL/6J mice at P1, P3, P5, and P10 (Fig. 2b). At P1 the PSS in both DBA/2J and C57BL/6J mice had clearly recognizable resting, proliferative, and hypertrophic chondrocyte zones, but the resting zone was narrower in DBA/2J mice (C57BL/6J: *M* = 165 μm, SD = 24 vs. DBA/2J: *M* = 129 μm, SD = 6, *p* < 0.01). By P5, there was a loss of distinct growth plate morphology in the PSS of DBA/2J mice; hypertrophic chondrocytes spanned the entire ventral surface of the synchondrosis (Fig. 2b, arrow). Interestingly, the PSS began to bulge dorsally, as if the direction of growth had changed from the normal bidirectional rostral–caudal axis to a unidirectional dorsal axis. By P10, the PSS was reduced to an indistinct mass of cartilage that projected dorsally and was ossified ventrally in DBA/2J mice (Fig. 2b).

### PSS closure does not follow a simple Mendelian inheritance pattern when DBA/2J and C57BL/6J mice are intercrossed

We crossed DBA/2J and C57BL/6J mice to determine whether PSS closure was heritable and, if so, to see if it was due to a single locus. Since we could see evidence of PSS closure in DBA/2J mice when their cranial bases were examined at P21, we decided to score all offspring at this age. Offspring were considered affected if under the dissecting microscope we observed superior protrusion of the PSS cartilage, angular deformity of the PSS, or unilateral or bilateral bony bridging. We performed crosses using male and female mice from both strains to control for parent-of-origin effects. None of the F1 offspring exhibited PSS closure (0/67). When we intercrossed F1 animals to produce F2 offspring, 17.9 % (86/481) had PSS closure. When we backcrossed F1 animals to DBA/2J mice, 52.9 % (45/85) of offspring had PSS closure. Although the observed segregation pattern of the backcross offspring did not differ significantly from the pattern expected for autosomal recessive inheritance (*p* = 0.59), this was not the case for the F1 intercross offspring (*p* = 0.00031), suggesting that the genetic control of the PSS closure trait is complex.

**Fig. 3:**
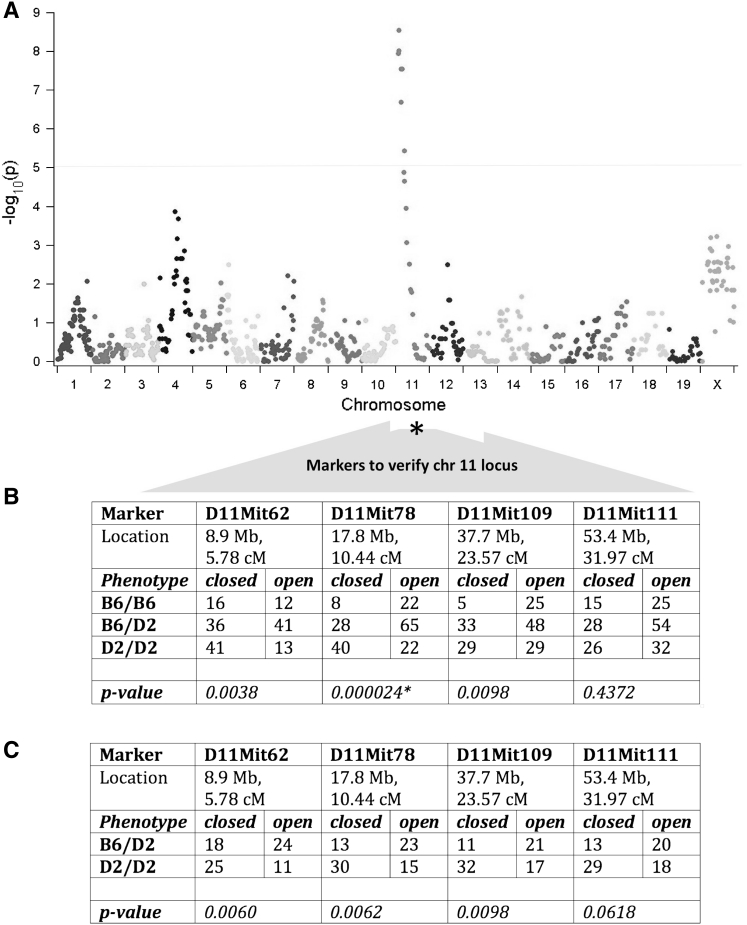
PSS closure is influenced by a locus on chromosome 11 but is not a simple Mendelian trait. **a** Manhattan plot depicting −log_10_ *p* values for 856 informative SNPs that were genotyped in 37 offspring from DBA/2J × C57BL/6J F1 intercross. All 37 offspring had exhibited PSS closure by P21. A *dotted horizontal line* (at −log_10_ *p* = 5) indicates the threshold for genome-wide significance. *p* values for several SNPs on proximal chromosome 11 exceed this threshold. **b** Table containing the frequencies of homozygous C57BL/6J genotypes (B6/B6), compound heterozygous C57BL/6J/DBA/2J genotypes (B6/D2), and homozygous DBA/2J genotypes (D2/D2) for microsatellite markers in a larger series of DBA/2J × C57BL/6J F1 intercross offspring that exhibited closure of the PSS (*closed*) or patency of the PSS (*open*) at P21. All offspring with PSS closure were genotyped, as were an equal number of randomly chosen offspring that had patent PSS. There was no enrichment for D2/D2 genotypes among offspring with open PSS, whereas D2/D2 genotypes were significantly enriched in offspring with PSS closure (*p* values represent the difference in the genotype distribution between offspring with closed and open PSS as determined by χ^2^ analysis). Significant *p* values were also obtained when the genotype distributions in offspring with closed PSS were compared with the null hypothesis (not shown). **c** Table containing the frequencies of compound heterozygous C57BL/6J/DBA/2J genotypes (B6/D2) and homozygous DBA/2J genotypes (D2/D2) for microsatellite markers F1 × DBA/2J backcross offspring that exhibited closure of the PSS (*closed*) or patency of the PSS (*open*) at P21. D2/D2 genotypes were significantly enriched in offspring with PSS closure (*p* values represent the difference in the genotype distribution between offspring with closed and open PSS as determined by χ^2^ analysis). Significant *p* values were also obtained when the genotype distributions in offspring with closed PSS were compared with the null hypothesis (not shown) (Color figure online)

We genotyped 37 F1 intercross offspring that had clear evidence of PSS closure to determine if a genetic locus could be identified that was consistent with autosomal recessive inheritance. We employed a high-density SNP array that contained 856 informative markers between DBA/2J and C57BL/6J mice. We found no evidence for simple Mendelian inheritance; instead, we observed highly significant enrichment for DBA/2J alleles on proximal chromosome 11 in the F2 mice with PSS closure (Fig. 3a). No other chromosomal region was significantly enriched, even after controlling for the chromosome 11 genotype (data not shown). In order to confirm the results of the SNP analysis, we used chromosome 11 microsatellite markers to genotype additional F2 animals with either opened or closed PSS (Fig. 3b). We observed a significant enrichment for DBA/2J alleles in offspring with closed PSS but not in offspring with open PSS. The DBA/2J allele for the microsatellite marker *D11Mit78* at 10.44 cM (located at 17.8 Mb on chromosome 11 in the mouse reference genome), exhibited the greatest enrichment in offspring with PSS closure. DBA/2J alleles were also enriched in offspring with closed PSS from the F1 × DBA/2J backcross (Fig. 3c). These results suggest that enrichment for DBA/2J alleles in this portion of chromosome 11 is related to PSS closure and is not due to other reasons.

### The age-dependent penetrance of PSS closure in the DBA/2J strain is influenced by alleles from the closely related DBA/1J strain

Among the many inbred strains, DBA/2J is most closely related to the DBA/1J strain, having been separated genetically 82 years ago. Therefore, we determined whether PSS closure also occurs in the DBA/1J strain. We observed no evidence of closure at P21 in 30 DBA/1J mice. We then crossed DBA/1J with DBA/2J mice to determine the inheritance pattern of PSS closure in their offspring. When we examined the F1 offspring at P21, 59 % (48/81) had PSS closure. When we examined F1 offspring at P35, 90 % (19/21) exhibited PSS closure. This result suggests that alleles inherited from the DBA/1J strain alter the timing of PSS closure. Consistent with this hypothesis, when we performed an F1 intercross and examined F2 offspring at P35, we observed PSS closure in 63 % (15/24) of the offspring.

**Fig. 4:**
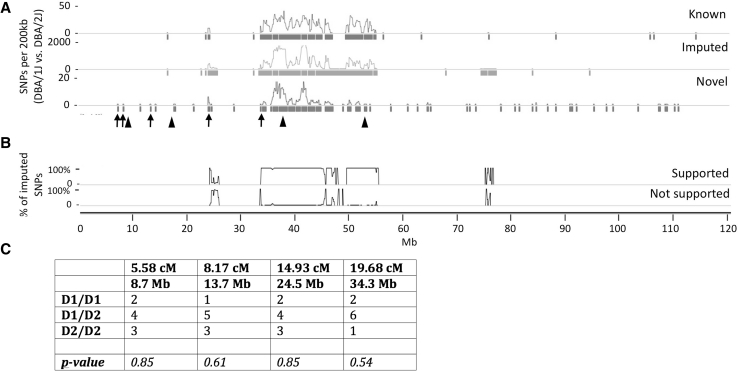
Chromosome 11 SNPs that distinguish the DBA/1J and DBA/2J strains. **a** Graph depicting the density (per 200-kb window) and distribution of SNPs that differ between the DBA/1J and DBA/2J strains across chromosome 11. Density tracings (SNPs/200 kb) represent previously known (*blue*), imputed (*gray*), and newly identified (*red*) SNPs. *Colored bars* below each density tracing are used to indicate when a 200-kb window contains at least one SNP. Locations of the SNPs and microsatellites that were used to genotype individual mice are indicated by *arrows* and *arrowheads*, respectively. **b** Graph depicting the percentage of imputed SNPs whose existence is supported (*upper*) or not supported (*lower*) by our data. When calculating these percentages, only SNPs covered by at least one read in both DBA/1J and DBA/2J were considered. **c** Table containing the frequencies of homozygous DBA/1J genotypes (D1/D1), compound heterozygous DBA/1J/DBA/2J genotypes (D1/D2), and homozygous DBA/2J genotypes (D2/D2) for SNPs in the nine P35 DBA/1J × DBA/2J F1 intercross offspring that still had an open PSS (Color figure online)

If a single locus in the DBA/1J strain was responsible for influencing PSS closure, F2 offspring with open PSS at P35 should be enriched for DBA/1J alleles at that locus. To identify genomic regions enriched for DBA/1J alleles, we performed low-coverage WGS on pooled DNA from the nine P35 F2 offspring that had an open PSS. Since the DBA/1J strain has not been fully sequenced, we also performed WGS on pooled DNA from two adult DBA/1J females. We obtained 76 million and 96 million 100-bp Illumina single-end reads, which resulted in 2.3- and 2.9-fold genome coverage when aligned to the reference genome (mm9), for DBA/1J and the F2 offspring, respectively. We compared our DBA/1J sequence to the publicly available DBA/2J sequence, which had also been aligned to the reference genome (Keane et al. 2011), to identify SNPs that distinguish the two strains. We first looked at the 19,933 SNPs that have previously been reported to distinguish the DBA/1J and DBA/2J strains. Our WGS covered 12,814 (68 %) of these sites and confirmed that most (98.8 %) were true SNPs (Supplementary Tables 1 and 2). We next looked at the 866,262 SNPs that were computationally predicted (i.e., imputed) to differ between DBA/1J and DBA/2J strains. Our WGS data covered 508,017 (58 %) of these imputed SNPs and confirmed that 91 % were true SNPs (Fig. 4a, b, Supplementary Fig. 1, Supplementary Tables 3 and 4). These results suggest that our low-coverage WGS is able to detect previously identified and predicted SNPs and, therefore, will detect novel SNPs. We were unable to confirm 1.2 % of the previously reported SNPs and 9 % of imputed SNPs, which were covered by our WGS data. SNPs that were not supported by our data may represent sequencing or alignment errors in our WGS, or errors in the SNP databases.

To identify novel SNPs that distinguish DBA/1J from DBA/2J, we required that the variant be present in at least two high-quality sequencing reads (see “Materials and methods” section for filtering criteria). We identified 142,935 SNPs, of which 92 % had previously been reported or imputed to distinguish DBA/1J from DBA/2J, while 11,194 (8%) had not (Supplementary Fig. 1). Of these newly identified differences between DBA/1J and DBA/2J, 77 % occurred at known mouse SNP sites, while 2,611 (23 %) were entirely new SNPs (Supplementary Tables 5 and 6). These latter SNPs likely arose as a consequence of mutation after the divergence of the DBA/1J and DBA/2J strains. Ten of these new SNPs are in coding regions (Supplementary Table 7). As would be expected for new mutations, the entirely new SNPs were distributed across the genome. We chose five SNPs that mapped to chromosome 11 for validation and confirmed that all were true SNPs.

Using a strategy we had successfully employed to map autosomal recessive traits in zebrafish using pooled DNA (Bowen et al. 2012), we scanned the WGS from pooled F2 offspring for regions enriched for DBA/1J alleles, using 10-Mb sliding windows. We did not detect any regions with a strong enrichment for DBA/1J alleles (Supplementary Fig. 2). However, the usefulness of this approach was limited due to regions of low SNP density between DBA/1J and DBA/2J (34 % of the genome contained no more than four SNPs per 10-Mb window). Therefore, we focused on the interval in chromosome 11 that was strongly associated with PSS closure in the DBA/2J × C57BL/6J cross. We determined genotypes for four SNPs across the chromosome 11 interval in the nine P35 F2 offspring with open PSS (Fig. 4a). There was no enrichment for DBA/1J alleles in this region (Fig. 4c), indicating that chromosome 11 in the DBA/1J strain does not modify the penetrance of PSS closure.

## Discussion

We report that the PSS closes in the DBA/2J strain and not in other common inbred strains. At the histologic level, differences between the PSS in the DBA/2J and C57BL/6J strains are detectable by P3. In contrast to the C57BL/6J strain in which the PSS diminishes in size but maintains its normal chondrocyte orientation as the mice age, the chondrocyte orientation appears to switch from rostral–caudal to dorsal in the DBA/2J strain. At present, we do not know whether this switch in orientation is intrinsic to the chondrocytes or the consequence of extrinsic mechanical forces that can result following the formation of a bony bridge across the structure.

In long-bone growth plates, proliferative chondrocytes have been demonstrated to divide orthogonal to the plane of growth and then intercalate to form columns that run parallel to the axis of growth (Li and Dudley 2009). Disruption of signaling pathways that orient chondrocyte division and intercalation has been shown to affect bone growth (Koziel et al. 2004; Viviano et al. 2005; Yang et al. 2003), raising the possibility that similar disruptions could affect PSS growth. In this regard, it is interesting that a primary cilia protein, PKD1/1, maps to mouse chromosome 11 and contains a nonsense mutation in the DBA/2J strain but not in any of the common inbred strains in which the PSS remains open. We also observed this nonsense mutation in our whole-genome sequencing data from the DBA/1J strain, which is consistent with PSS closure being observed in DBA/1J × DBA/2J F1 offspring. Although this mutation is intriguing, it and other coding and noncoding variants that will be common to DBA/1J and DBA/2J on chromosome 11 cannot fully explain the occurrence of PSS closure; several F2 offspring from the DBA/2J × C57BL/6J F1 intercross with PSS closure did not inherit DBA/2J alleles on chromosome 11, and by P21, when most DBA/2J mice have begun closing their PSS, no evidence of closure is seen in the DBA/1J strain.

Two known genes lie near the microsatellite marker *D11Mit78* at 10.44 cM, which exhibited the greatest enrichment in DBA/2J alleles in offspring with PSS closure. *DGVi11a* epigenetically modulates *HipV13a*, affecting dentate gyrus volume (Peirce et al. 2003). Another gene, *Etaa1*, is homologous to *ETAA1* (Ewing tumor-associated antigen 1; synonym: *ETAA16*) in humans, which is expressed on bone tumor cells of mesenchymal origin or sarcomas (Borowski et al. 2006). There are also two unknown or predicted genes near *D11Mit78*, *Gm12016* and *2900053O20Rik*.

By performing low-coverage whole-genome sequencing of the DBA/1J strain, we were able to identify more than 11,000 SNPs that had not previously been known to distinguish DBA/1J from DBA/2J. These SNPs were distributed throughout the genome, including regions of extremely high sequence conservation between the DBA/1J and DBA/2J strains. This strongly indicates that these variants arose after the strains were separated. These novel variants will be useful for genotyping intercross offspring until the entire SNP repertoire within the DBA/1J strain is determined by deep sequencing approaches. For example, we used novel SNPs identified by WGS to exclude chromosome 11 in the DBA/1J strain from harboring a strong modifier locus for age-dependent PSS closure in F1 intercross offspring.

We cannot exclude the possibility that PSS closure observed in the DBA/2J mice is a consequence of indirect effects on the PSS. Histologic examination of the early stages of closure reveal ventral and lateral bony bridges forming around the same time that chondrocytes within the growth plate change their orientation. Therefore, chondrocyte reorientation and PSS closure could be the consequence of abnormal ossification rather than the cause. Others have shown that external mechanical forces are able to induce changes in chondrocyte differentiation (Lei et al. 2008).

Our studies indicate that PSS closure in the DBA/2J strain does not follow a simple pattern of inheritance but that closure is strongly influenced by allele(s) on mouse chromosome 11. In humans, the intrasphenoidal synchondrosis corresponds to the PSS in mice. Closure of the intrasphenoidal synchondrosis normally occurs shortly after birth. Therefore, the DBA/2J strain, whose PSS also closes shortly after birth, may be a useful model for understanding molecular mechanisms that contribute to normal synchondrosis closure in humans.

## Electronic supplementary material

**Below is the link to the electronic supplementary material.**

**Supplementary Fig. 1** **(PDF 6160 kb)**

**Supplementary Fig. 2** **(PDF 1836 kb)**

**Supplementary Table 1** **(TXT 234 kb)**

**Supplementary Table 2** **(TXT 3 kb)**

**Supplementary Table 3** **(TXT 8558 kb)**

**Supplementary Table 4** **(TXT 860 kb)**

**Supplementary Table 5** **(TXT 176 kb)**

**Supplementary Table 6** **(TXT 54 kb)**

**Supplementary Table 7** **(TXT 2 kb)**
