# Supplementary material for: Presphenoidal synchondrosis fusion in DBA/2J mice
Source: Mamm Genome. 2012 Nov 21;24(1):54–62. doi: 10.1007/s00335-012-9437-8 (PMC3560942; doi:10.1007/s00335-012-9437-8)
Supplement: Supplementary file 1 — Supplementary Fig. 1 (PDF 6160 kb) [file 335_2012_9437_MOESM1_ESM.pdf]

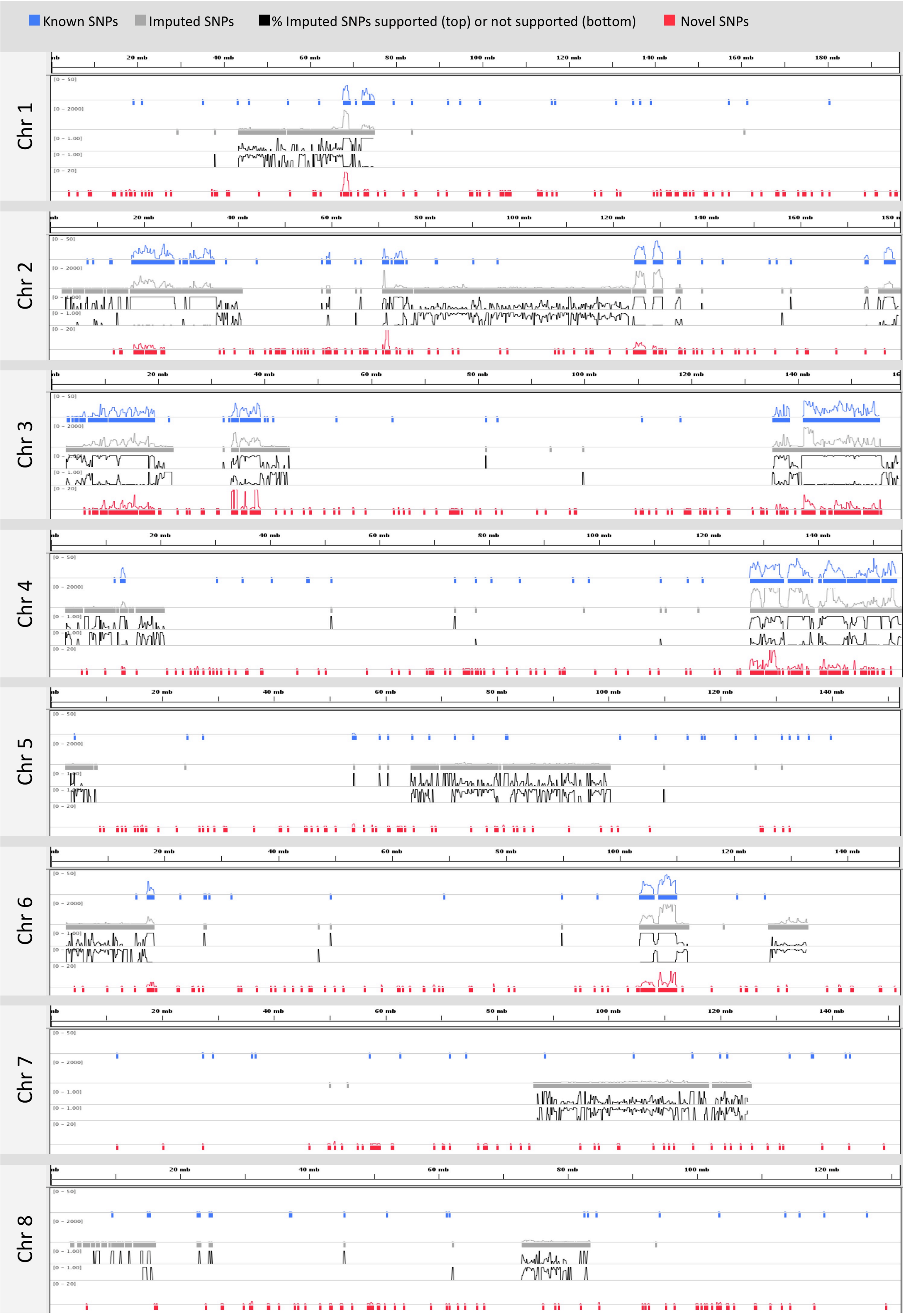

Known SNPs
Imputed SNPs
% Imputed SNPs supported (top) or not supported (bottom)
Novel SNPs
Chr 1
Chr 2
Chr 3
Chr 4
Chr 5
Chr 6
Chr 7
Chr 8

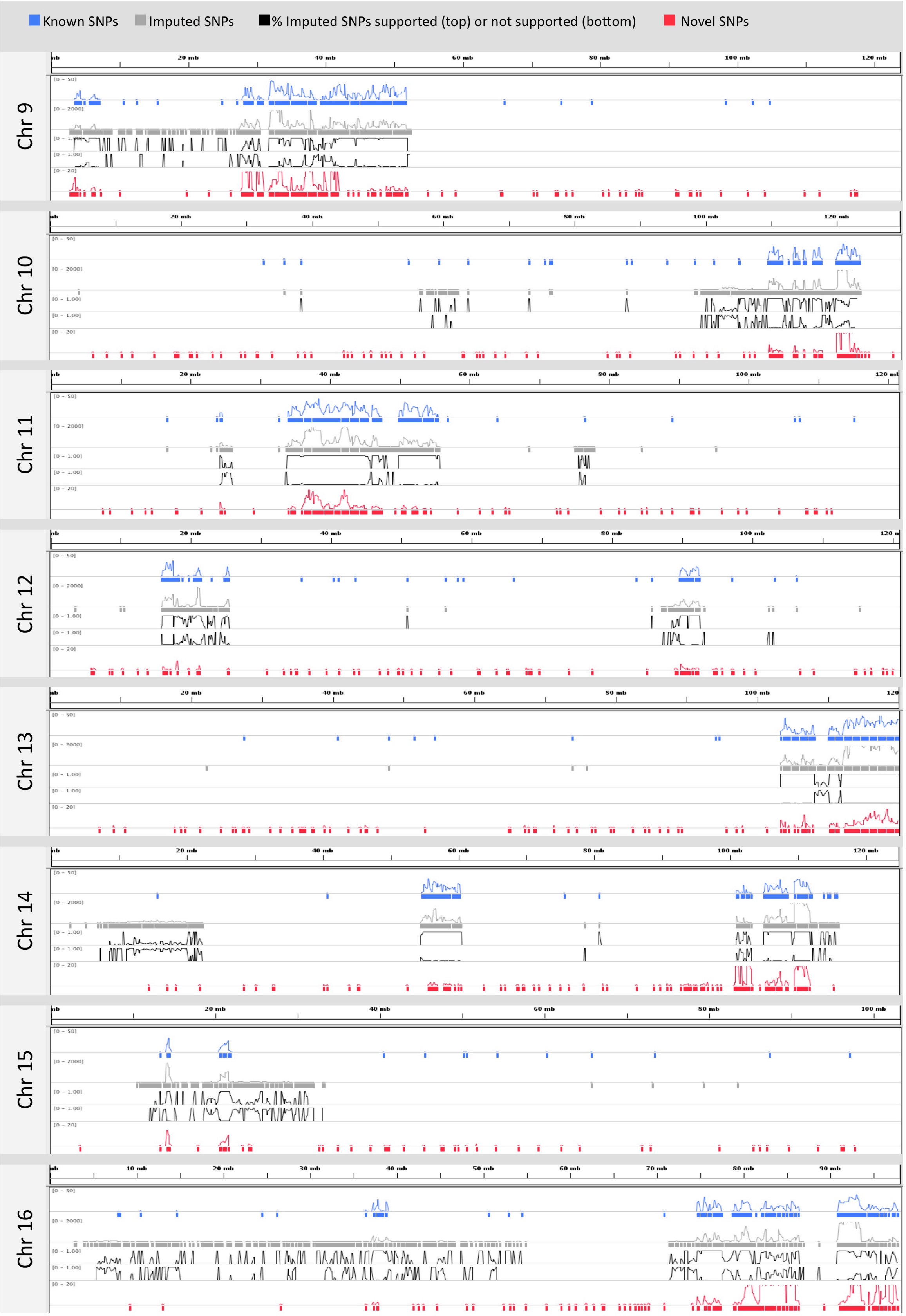
Known SNPs
Imputed SNPs
% Imputed SNPs supported (top) or not supported (bottom)
Novel SNPs
Chr 9
Chr 10
Chr 11
Chr 12
Chr 13
Chr 14
Chr 15
Chr 16

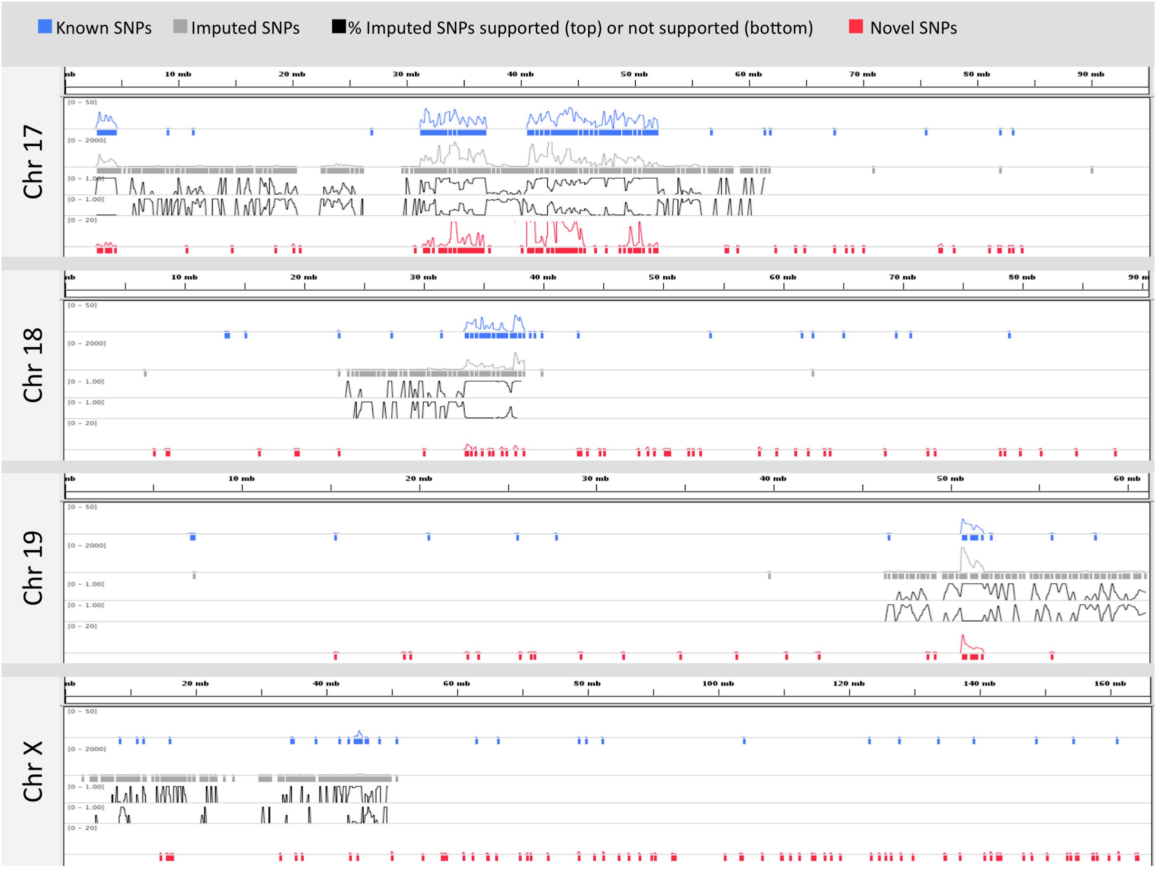

Supplementary Figure 1: Genomic distribution of SNPs that distinguish the DBA/1J and DBA/2J strains. Graphs depicting the density (per 200kb window) and distribution (along each chromosome) of SNPs that differ between the DBA/1J and DBA/2J strains. Density tracings (SNPs/200kb) represent previously known (blue), imputed (gray), and newly identified (red) SNPs. Colored bars below each density tracing are used to indicate when the 200 kb window contains at least 1 SNP. The 2 tracings in black indicate the percentage of imputed SNPs whose existence is supported (upper) or not supported (lower) by our data. When calculating these percentages, only SNPs covered by at least one read in both DBA/1J and DBA/2J were considered. The scale of the y-axis is indicated in the top left corner of each tracing.
