# Supplementary material for: Presphenoidal synchondrosis fusion in DBA/2J mice
Source: Mamm Genome. 2012 Nov 21;24(1):54–62. doi: 10.1007/s00335-012-9437-8 (PMC3560942; doi:10.1007/s00335-012-9437-8)
Supplement: Supplementary file 2 — Supplementary Fig. 2 (PDF 1836 kb) [file 335_2012_9437_MOESM2_ESM.pdf]

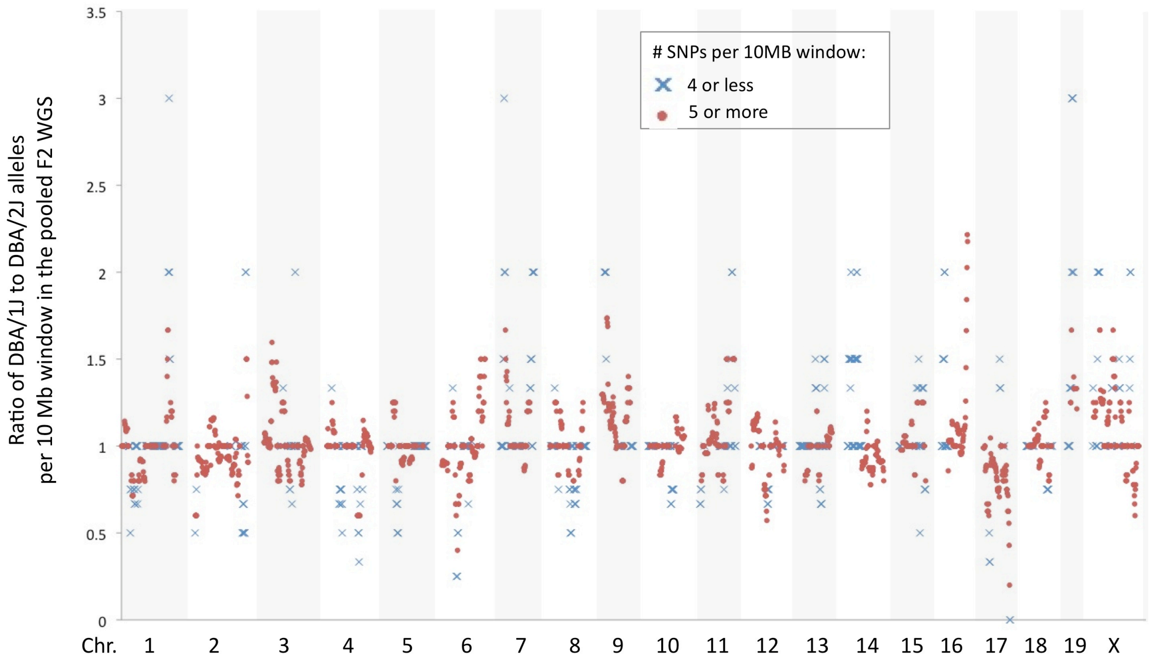

Supplementary Figure 2: Scanning the genome for regions enriched in DBA/1J alleles. Graph showing the ratio of DBA/1J alleles to DBA/2J alleles observed in the pooled F2 offspring WGS. Ratios are calculated for sliding windows of 10 Mb in size, with an overlap of 1 Mb between adjacent windows. Only SNP positions at which the DBA/1J and DBA/2J strains were each homozygous for different alleles were considered. SNP sites in the pooled F2 offspring were counted as having the DBA/1J allele if at least one sequencing read at that site represented the DBA/1J allele, and vice versa. Windows with only 1 or zero SNP sites (7%) were excluded from the analysis, windows with 2 to 4 SNPs (27%) are shown with blue crosses, and windows with 5 or more SNPs (66%) are shown in red circles. As expected, most windows have a ratio of ~1, indicating an equal representation of DBA/1J and DBA/2J alleles. Note that no windows have a ratio greater than 3:1. Therefore, there is no evidence for a strong enrichment for DBA/1J alleles in any genomic region. Similar results were obtained if a smaller window size (2 Mb) or larger window size (20 Mb) were used.
